# The Effectiveness of Online Versus Group Prenatal Education on Breastfeeding Determinants and Outcomes in Nulliparous Women: A Prospective Cohort Study

**DOI:** 10.1111/jmwh.70156

**Published:** 2026-07-07

**Authors:** Marie‐Violaine Ponte, Alexandre Prud'homme, Sonia Semenic, Stéphane Turcotte, Maude Laberge, Nils Chaillet, Geneviève Roch

**Affiliations:** ^1^ Centre de recherche du CHU de Québec–Université Laval Québec Québec Canada; ^2^ VITAM – Centre de recherche en santé durable Québec Québec Canada; ^3^ Ingram School of Nursing McGill University Montreal Québec Canada; ^4^ Centre de recherche du CISSS de Chaudière‐Appalaches Lévis Québec Canada; ^5^ Faculty of Medicine Université Laval Québec Québec Canada; ^6^ Faculty of Nursing Université Laval Québec Québec Canada

**Keywords:** breastfeeding, education, distance, maternal health services, prenatal education

## Abstract

**Introduction:**

Prenatal education is key to promoting breastfeeding. To address equity and access challenges, health care facilities are increasingly offering online prenatal education in addition to in‐person sessions. This study aimed to estimate the influence of online and group prenatal education services, used separately or together, on breastfeeding determinants (intentions, self‐efficacy) and outcomes (initiation, exclusivity) among nulliparous pregnant persons, compared with standard prenatal care.

**Methods:**

A prospective cohort study was conducted from June 2018 to September 2020 in Québec, Canada with 863 nulliparous pregnant persons from 2 adjacent regions. Prior to the COVID‐19 pandemic, 358 participants completed questionnaires at 3 time points about their use of prenatal education services in which breastfeeding‐related content was optional. Based on their self‐reported use, participants were divided into 5 categories: (1) face‐to‐face group prenatal education, (2) asynchronous online prenatal education, (3) both (with breastfeeding), (4) either group/online/both (opted out of breastfeeding content), and (5) standard prenatal care only. Repeated‐measures analysis of variance, generalized linear mixed models, mixed‐effects logistic regression, χ^2^, and Fisher's exact test were conducted.

**Results:**

A total of 53.3% of participants chose the prenatal education services with breastfeeding information. When compared with standard prenatal care only, exposure to breastfeeding information from the online option alone or from both services was associated with breastfeeding intentions and initiation. No prenatal education services were associated with self‐efficacy or exclusivity; however, higher prenatal breastfeeding self‐efficacy predicted exclusivity 4 weeks after childbirth.

**Discussion:**

Study findings highlight the complementary contribution of asynchronous online and in‐person group prenatal education services to breastfeeding, adding to the scant literature comparing these educational options. This can help health care providers, decision‐makers, and policy makers identify avenues to strengthen equity in the administration of breastfeeding education, thereby providing expectant persons with better strategies for choosing and maintaining breastfeeding.

## INTRODUCTION

Breastfeeding is promoted worldwide because of its maternal and infant health benefits,^1^ including protecting infants against gastrointestinal and respiratory infections, enhancing their cognitive development, and reducing maternal breast and ovarian cancer rates.^2^ The US and Canadian recommendations are to exclusively breastfeed all infants for 6 months.^3^, ^4^ Despite these guidelines, breastfeeding rates in North America fall rapidly in the first weeks after birth. Only 38.2% of infants in Canada^5^ and 25.4% in the United States^6^ are exclusively breastfed for the recommended 6‐month duration.

Although breastfeeding choices and behaviors reflect a combination of structural, environmental, and individual factors, prenatal education services play a pivotal role in promoting breastfeeding.^7^ In Québec, Canada, standard prenatal follow‐up care, comprising 10 and 13 in‐person appointments with registered nurses and physicians, nurse practitioners, or midwives, is available for free. In addition, many health care institutions offer optional prenatal education classes that cover topics related to pregnancy, birth, and infant care, including breastfeeding. To meet evolving parents’ needs and promote equitable access, some have been offering online asynchronous prenatal education (OPE) since 2015 while maintaining face‐to‐face group prenatal education (GPE) services. The GPE and OPE curriculum design are intended to provide the requisite information to make informed decisions on various perinatal topics.

1 **QUICK POINTS**

✦ More than half of participants (53.3%) engaged in prenatal education services with breastfeeding information, choosing asynchronous online prenatal education over other modalities.
✦ Exposure to breastfeeding information from both face‐to‐face group prenatal education and asynchronous online prenatal education services was associated with breastfeeding intentions and initiation among women who are nulliparous.
✦ Using group or online prenatal education was not associated with end‐of‐pregnancy breastfeeding self‐efficacy or breastfeeding exclusivity 4 weeks after childbirth among women who are nulliparous, compared with standard prenatal care only.
✦ Exclusive breastfeeding 4 weeks after childbirth was predicted by prenatal breastfeeding self‐efficacy.
✦ There remains a need to further promote and support access to various prenatal breastfeeding education services.

### Prenatal Breastfeeding Education

Prenatal educational interventions can improve knowledge and attitudes related to breastfeeding.^8^ particularly among expectant first‐time parents.^9^, ^10^ Educational interventions that foster performance accomplishments, vicarious experiences (or role‐modeling), verbal persuasion, and physiologic/affective state can increase breastfeeding self‐efficacy (BSE), a modifiable factor associated with positive breastfeeding outcomes.^11^, ^12^, ^13^, ^14^, ^15^, ^16^ However, it remains challenging to determine the specific impact of educational sources on breastfeeding behaviors. Most structured interventions designed to promote breastfeeding focus on the postnatal period.^17^, ^18^ Studies of the effectiveness of breastfeeding educational programs often recruit persons who intend to breastfeed, targeting those who are both nulliparous and multiparous.^19^, ^20^, ^21^ Moreover, they often fail to acknowledge that parents can access a multitude of alternative sources of breastfeeding information (eg, relatives, health care providers, the media, and digital applications) alongside standardized prenatal care.

The impact of GPE on breastfeeding has been compared with individual educational interventions, postnatal interventions, other specific nondigital modalities, or a combination of prenatal classes, one‐to‐one counseling sessions, and postnatal coaching.^17^, ^22^, ^23^ However, in real‐life settings, in which parents‐to‐be have the choice to engage or not in prenatal education, the distinctive contributions of OPE and GPE in fostering positive breastfeeding attitudes and behaviors remain to be clarified.

The purpose of this study was to estimate the relative effectiveness of asynchronous online and face‐to‐face GPE services, with optional breastfeeding‐related content, used either separately or together, on breastfeeding intention, self‐efficacy, and outcomes among first‐time mothers, compared with no use of prenatal education services (standard prenatal care follow‐up only).

### Hypotheses

Based on existing literature and theoretical assumptions,^16^, ^24^ we hypothesized that vicarious experiences and verbal persuasion^16^ received through the breastfeeding information presented in OPE or GPE, or both could positively enhance BSE. Following Donabedian's structure‐process‐outcome model for assessment of health care services,^24^ we also hypothesized that breastfeeding knowledge and skills acquired by women who are nulliparous through using these prenatal education modalities would significantly increase rates of breastfeeding intention, breastfeeding initiation, and exclusive breastfeeding at 4 weeks.

## METHODS

We tested our hypotheses in a prospective cohort study nested within a multicenter prospective cohort investigation.^25^ This larger project aimed to assess the impacts of group and online prenatal education services on various health determinants and users’ health status.

The Multicenter Ethics Committee of the Centre intégré de santé et de services sociaux de Chaudière‐Appalaches (MP‐23‐2017‐405) approved this study, which complies with the Declaration of Helsinki. We reported the findings in accordance with the Strengthening the Reporting of Observational Studies in Epidemiology Statement guidelines for cohort studies^26^ (Supporting Information: Appendix S1). DeepL Write (DeepL SE) was used to assist with editing for grammar and clarity. We subsequently reviewed all artificial intelligence–generated content.

### Study Setting

We recruited our sample from 2 adjacent regions (A and B) in the province of Québec, Canada that have a combined population of more than 1,000,000. Approximately 90% of the region A population are urban dwellers, whereas around 40% of the region B population reside in a rural area.

### Description of Interventions

GPE services were provided through in‐person group sessions organized by health care professionals from local health care and social service centers and birthing centers managed by regional health care boards. GPE sessions took place in 23 community health care centers and 2 birthing centers, were free of charge in one region, and had a maximum cost of $50 in the other (depending on participants' needs). Participants attended the number of GPE sessions of their choice, according to their needs. GPE and OPE services had in common the following content areas: (1) pregnancy and prenatal lifestyle, (2) labor and birth, (3) pain management during labor and birth, (4) obstetric procedures (eg, episiotomy, caesarean section, forceps), (5) breastfeeding, (6) postnatal physical and mental health of parents and newborn, (7) newborn care and parenting, and (8) community services and resources.

Nurses and midwives led the GPE sessions, and lactation consultants from breastfeeding support groups often joined them for breastfeeding‐oriented sessions. Each GPE session lasted from 120 to 150 minutes. The number of sessions offered ranged from 2 to 6 in one region, where only 6 of 16 sites collaborated with breastfeeding support groups. There was no explicit theoretical/pedagogical approach used, and teaching material varied from one site to another, although educational content was in the process of being harmonized across the region. Variations in the number of sessions and instructors influenced both the range of topics covered and the time allocated to each. For breastfeeding, the sessions ranged from 30 to 150 minutes in one region (mean 103 minutes) and from 75 to 240 minutes in the other (mean 144 minutes). For both GPE and OPE, engagement in the different sessions was optional for all participants.

To access the OPE sessions, participants used a single, free, web‐based educational platform managed by philanthropic providers with no commercial interests. To ensure alignment with new scientific evidence, a clinical expert review process guided regular updates of the platform's educational content. The content included various formats, such as headlines, podcasts, articles, and texts with read‐aloud features. Participants had the opportunity to download supplementary information that best suited their needs, including pictures, charts, and graphics. They were encouraged to sign up for a weekly, personalized email newsletter with information based on their expected due date. Further details on each prenatal education service are provided in Supporting Information: Appendix S2.

### Sample and Data Collection

We recruited participants using convenience sampling from June 2018 to December 2019, during routine ultrasound examinations in the hospital or prenatal follow‐up visits in medical clinics (through research staff) or clinical visits to local community health care centers (through health care providers), via social media (Facebook), and through public advertising in the recruitment sites. Figure 1 is the cohort study flow chart that summarizes the research process, and Figure 2 presents the average and total follow‐up times.

**Figure 1:**
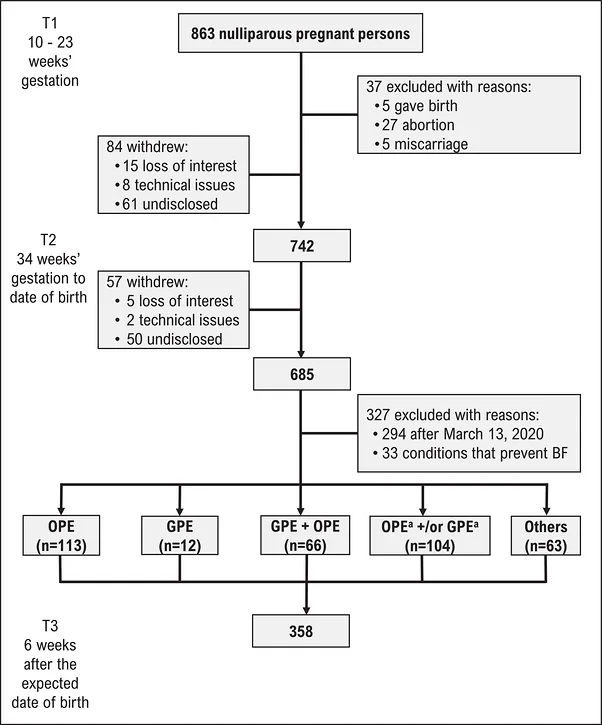
Flow Chart Abbreviations: BF, breastfeeding, GPE, face‐to‐face group prenatal education; OPE, asynchronous online prenatal education; T1, first questionnaire filled out by participants when recruited (10‐23 weeks’ gestation); T2, second questionnaire sent to the participants at the end of their pregnancy (34 weeks’ gestation: date of birth); T3, third questionnaire sent to participants 6 weeks after their expected date of birth. ^a^Opted out of breastfeeding content.

**Figure 2:**
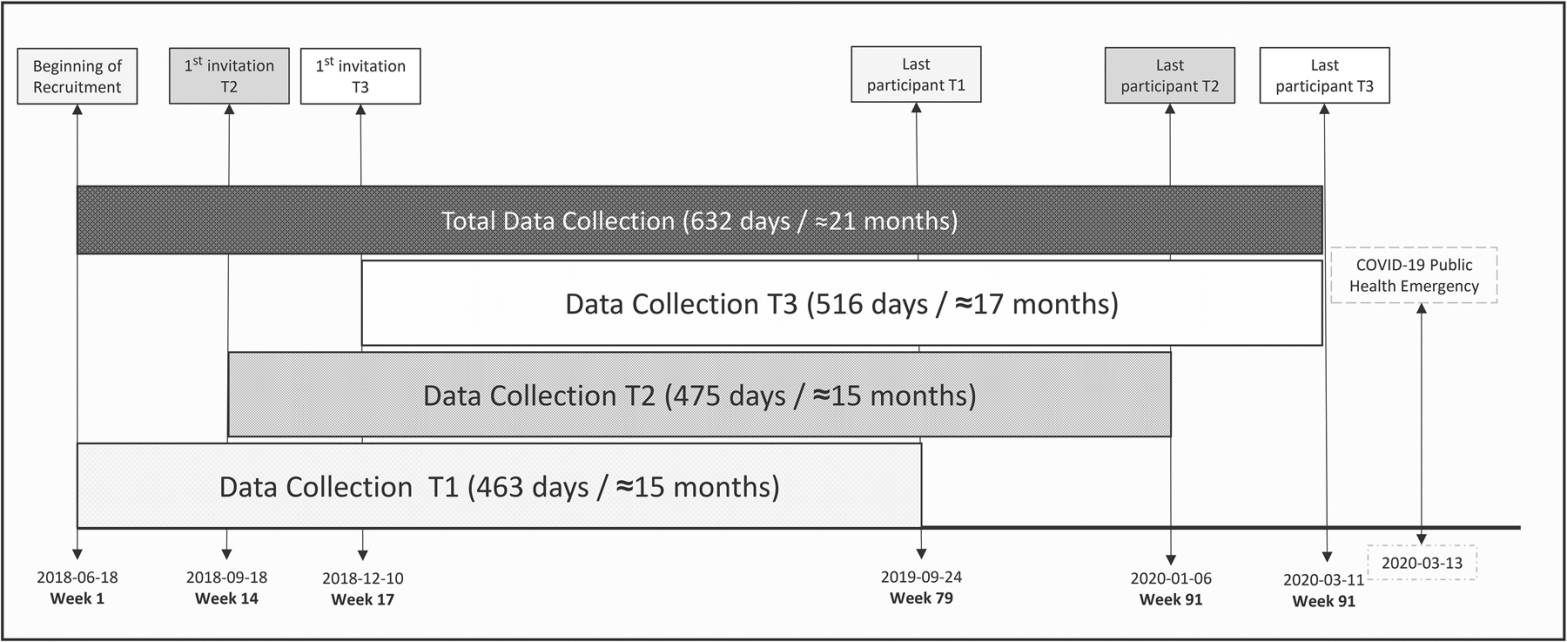
Follow‐up Time Abbreviations: T1, first questionnaire filled out by participants when recruited (10‐23 weeks’ gestation); T2, second questionnaire sent to the participants at the end of their pregnancy (34 weeks’ gestation: date of birth); T3, third questionnaire sent to participants 6 weeks after their expected date of birth.

Participants were eligible for the study if they were adults (≥18 years old), were between 10 and 23 weeks pregnant, were nulliparous, were registered to receive GPE or OPE services in one of the 2 target regions, understood French, and had a valid email address and internet access.^25^ We excluded those who presented with a condition that would interfere with breastfeeding or completed the postnatal questionnaire after March 13, 2020, to overcome bias introduced by changes to the provision of perinatal services because of Québec's COVID‐19 public health emergency safety measures.

For this nested cohort, we collected data from June 2018 to March 2020. We explained the objectives and nature of the study to potential participants, and those interested signed a detailed informed consent form. We sent email invitations to potential participants who met the eligibility requirements to fill out the first of 3 online questionnaires. We collected data related to breastfeeding and prenatal education at 3 time points: T1 (10‐23 weeks’ gestation), T2 (34 weeks’ gestation—date of birth), and T3 (4 weeks after expected date of birth). At each time point, we sent up to 2 reminder emails and made a phone call to participants who had not yet filled out the questionnaire. For each completed questionnaire, we entered participants into a draw to win a tablet computer. We provided each participant who completed the third questionnaire with a $10 gift card from a local business.

### Data Analysis

#### Variables of Interest

The main independent variable was the use of prenatal education services. Outcomes variables included: (1) BSE, (2) intentions to breastfeed, (3) initiation of breastfeeding, and (4) exclusive breastfeeding at 4 weeks postpartum.

##### Use of Prenatal Education Services

At the end of their pregnancy (T2), participants were asked about their use of prenatal education services. For the independent variable, we separated our sample as follows: (1) GPE only (face‐to‐face GPE; with breastfeeding information), (2) OPE only (OPE; with breastfeeding information), (3) both GPE and OPE (with breastfeeding information), (4) GPE and OPE (opted out of breastfeeding content), and (5) no prenatal education (standard prenatal care only) (Table 1). The study design assumes that participants may have received breastfeeding information from family, friends, health care professionals, mobile applications, other prenatal education services (eg, private prenatal education sessions), or other sources of prenatal information in addition to those offered by their regional institutions.

**Table 1:** Description of PE Services Categories According to Participants’ Self‐Reported Use

**GPE Only (With Breastfeeding Information)**
Attended face‐to‐face GPE
No asynchronous OPE
Decided on receiving breastfeeding‐related content
Standard prenatal care by MDs, RNs, or midwives
**OPE Only (With Breastfeeding Information)**
Used OPE
No GPE
Reported accessing OPE breastfeeding‐related content
Standard prenatal care by MDs, RNs, or midwives
**GPE and OPE (With Breastfeeding Information)**
Attended face‐to‐face GPE
Used asynchronous OPE
Decided on receiving/accessing GPE/OPE breastfeeding‐related content
Standard prenatal care by MDs, RNs, or midwives
**GPE and OPE (Opted Out of Breastfeeding Content)**
Either enrolled in face‐to‐face GPE and used asynchronous OPE
Did not enroll in sessions with/did not access breastfeeding‐related content
**No PE (Standard Prenatal Care Only)**
Received standard prenatal care follow‐up without enrolling in OPE or GPE

Abbreviations: GPE, face‐to‐face group prenatal education; MD, doctor of medicine; OPE, online asynchronous prenatal education; PE, prenatal education; RN, registered nurse.

##### BSE

We assessed BSE with a French version of the Breastfeeding Self‐Efficacy Scale—Short Form (BSES‐SF),^28^ a valid and reliable tool that can be used prenatally^29^ to evaluate ones’ confidence in their ability to breastfeed^30^ (Cronbach's α =  .94). The BSES‐SF is a 14‐item questionnaire using a 5‐point Likert‐type scale ranging from 1 (not at all confident) to 5 (always confident). Total scores range from 14 to 70, with higher scores indicating higher BSE. Participants were sent the BSES‐SF at each time point unless they declared no intention to breastfeed (T1, T2) or no breastfeeding initiation (T3). To facilitate understanding of the BSES‐SF in Québec, we reworded some items slightly; minor alterations to the wording of the T1 BSES‐SF ensured suitability for use in a prenatal context (Cronbach's α: T1 =  .944, T2 =  .951, T3 =  .968).

##### Intention to Breastfeed

We assessed intention to breastfeed at T1 and T2, through the question: “Are you considering breastfeeding?” The possible answers were (1) yes, (2) no, and (3) I don't know/I don't want to answer.

##### Initiation of Breastfeeding

We assessed initiation of breastfeeding (expressed milk or direct breastfeeding) at T3 through the question: “Have you breastfed your baby since birth?” The possible answers were (1) yes, (2) no, and (3) I don't want to answer. Participants who answered “no” were asked an open‐ended question to specify how their infant had been fed since birth. Those who mentioned expressed milk were merged with those who answered “yes.”

##### Exclusive Breastfeeding at 4 Weeks Postpartum

To address our study's objectives, we asked 5 questions at T3 on infant feeding behaviors, organized into 4 categories: exclusive breastfeeding (breastmilk, from the breast or expressed, without any other liquids or solids, with the exception of drops/syrups/vitamins/oral rehydration solution),^31^ nonexclusive breastfeeding (eg, breastfeeding and infant formula feeding), infant formula feeding with previous breastfeeding, and exclusive infant formula feeding.^32^, ^33^ Because public health care recommendations target exclusive breastfeeding rates, results were dichotomized into exclusive breastfeeding versus other types of infant feeding. In Canada, the largest decline in breastfeeding rates occurs during the first month after childbirth, which underscores the first 4 weeks postpartum as a critical period for breastfeeding success.^34^, ^35^

#### Sociodemographic and Other Confounding Variables

Several sociodemographic variables and psychosocial determinants have been reported to influence breastfeeding.^30^, ^36^, ^37^, ^38^, ^39^, ^40^, ^41^ The following control variables were therefore assessed: prenatal generalized anxiety, prenatal BSE, maternal age, average household income, country of birth, level of education, marital status, and region of residency.

#### Analyses

We conducted all data analyses using IBM SPSS (version 29.0). Statistical level of significance was set at 5%. We expressed descriptive statistics as absolute (n) and relative (%) frequencies for categorical variables, and as means and SDs or medians and IQRs for continuous variables. We examined distributions using the Kolmogorov‐Smirnov test and graphical methods.

We performed a logarithmic transformation of the BSES‐SF scores because they showed a bimodal distribution at each time point. We used a 2‐way analysis of variance for repeated measures (prenatal education services × time [T1, T2, T3]) (general linear mixed model) to assess the influence of prenatal education services on BSE.^32^

We performed a general linear model for repeated measures (prenatal education services × time [T1, T2]) using generalized estimating equations (GEEs)^33^ to assess changes in participants’ breastfeeding intentions.

We performed a logistic regression to estimate the multivariate associations with prenatal education services and breastfeeding initiation (T3) or exclusive breastfeeding at 4 weeks postpartum (T3). Residuals were analyzed, and the area under the curve and the Hosmer‐Lemeshow test were used to assess the conformity of the analyses.

### Handling of Missing Data

Participants had to reply to all questions with the option of “I don't know” or declining to answer. To construct the outcome variables, we imputed values to all “Refusals” or “I don't know” responses. We performed a probabilistic imputation for each item.^42^ Missing data never exceeded 5% of the raw distribution.

## RESULTS

### Characteristics of the Sample

A total of 863 women who were nulliparous participated in the overall cohort investigation. However, 364 participants completed their data collection after the start of Québec's pandemic lockdown (n = 294) and had conditions that would interfere with breastfeeding (n = 33), and 141 were lost to follow‐up (Figure 1), resulting in a final sample of 358 participants. For the analysis of breastfeeding exclusivity at 4 weeks, we excluded 2 participants who completed the postnatal questionnaire (T3) when their newborns were one and 3 weeks old.

Table 2 presents participants’ individual characteristics. The mean age of participants was 29.1 (SD, 4.2) years, with the largest age group being those between 25 and 29 years (46.1%; n = 165). Most participants had a university degree (n = 221, 61.7%) and an annual household income higher than $100,000 (n = 159, 44.4%). Most participants lived with a partner (n = 325, 90.8%). Regarding prenatal education services, 53.3% of participants used prenatal education services with breastfeeding information, choosing OPE more often (n = 113, 31.6%), whereas 12 (3.4%) used GPE solely.

**Table 2:** Baseline Characteristics of Participants (n = 358)

Characteristics	n (%)
**Parental age, y**	
18‐24	43 (12.0)
25‐29	165 (46.1)
30‐34	110 (30.7)
>35	40 (11.2)
**Educational level**	
High school or less	59 (16.5)
College	78 (21.8)
University	221 (61.7)
**Household gross annual income**	
<$40,000	37 (10.3)
$40,000‐$59,000	36 (10.1)
$60,000‐$79,000	66 (18.4)
$80,000‐$99,000	60 (16.8)
>$100,000	159 (44.4)
**Marital status**	
Single/separated/divorced	33 (9.2)
Common law/married	325 (90.8)
**Country of birth**	
Other than Canada	23 (6.4)
Canada	335 (93.6)
**Region of residence**	
A	60 (16.8)
B	298 (83.2)
**PE services**	
GPE and OPE (with breastfeeding information)	66 (18.4)
GPE only (with breastfeeding information	12 (3.4)
OPE only (with breastfeeding information)	113 (31.6)
GPE and OPE (opted out of breastfeeding content)	104 (29.0)
No PE (standard prenatal care only)	63 (17.6)

Abbreviations: GPE, face‐to‐face group prenatal education, OPE, online asynchronous prenatal education; PE, prenatal education.

### BSE

The generalized linear model indicates that throughout the 3 time points, neither the use of OPE nor GPE was associated with the BSE of women who were nulliparous (Table 3). Terms of interaction were tested but did not change the results.

**Table 3:** Associations Between Prenatal Education Services or Time Point and BSE (Generalized Linear Mixed Models^a^)

Parameter	β (95% CI)	*P* Value
**Time point** ^b^		
T1	Reference	
T2	.06 (0.02 to 0.10)	.005
T3	.06 (0.01 to 0.1)	.014
**Prenatal education services** ^b^		
No PE (standard prenatal care only)	Reference	
GPE and OPE (with breastfeeding information)	.07 (0.01 to 0.13)	.027
GPE only (with breastfeeding information)	.04 (−0.15 to 0.06)	.429
OPE only (with breastfeeding information)	.02 (−0.03 to 0.07)	.459
GPE and OPE (opted out of breastfeeding content)	−.002 (−0.06 to 0.05)	.935

Abbreviations: BSE, breastfeeding self‐efficacy; df, degree of freedom; GPE, face‐to‐face group prenatal education, OPE, asynchronous online prenatal education; T1, 10‐23 weeks’ gestation; T2, 34 weeks’ gestation (date of birth); T3, 6 wk after the expected date of birth.

a Adjusted for prenatal general anxiety, age, educational attainment, household gross annual income, marital status, and country of birth.

b Tests of model effects on BSE scores were statistically significant for time (Wald χ^2^, 9.35 [df = 2]; *P =  *.009) but not for prenatal education services. This must be considered when interpreting the single effects displayed.

### Intention to Breastfeed

Regardless of the educational modality, breastfeeding intention rates increased from 79.5% at T1 to 84.4% at T2. The GEE model (Table 4) suggests that participants with intentions to breastfeed at T1 were more likely to maintain their intention at T2 (*P* =  .006) and that a significantly higher intention to initiate breastfeeding was more likely among participants using OPE (β = 2.652; 95% CI, 1.308‐5.375; *P =  *.007) or both OPE and GPE (β = 9.296; 95% CI, 2.895‐29.847; *P* <  .001) compared with standard prenatal care. The overall GEE tests of model effects were statistically significant (prenatal education services: Wald χ^2^, 21.536; *P <*  .001; time: Wald χ^2^, 7.463; *P <*  .006).

**Table 4:** Associations Between PE Services or Time Point and Intention to Breastfeed (Generalized Estimating Equations^a^)

Parameter	OR (95% CI)	*P* Value
**PE services**		
No PE (standard prenatal care only)	1 (reference)	
GPE and OPE (with breastfeeding information)	9.3 (2.9‐29.9)	<.001
GPE only (with breastfeeding information)	1.42 (0.35‐5.74)	.62
OPE only (with breastfeeding information)	2.65 (1.31‐5.38)	.007
GPE and OPE (opted out of breastfeeding content)	1.08 (0.56‐2.11)	.812
**Time point** ^b^		
T1	1 (reference)	
T2	1.37 (1.09‐1.727)	.006

Abbreviations: df, degree of freedom; GPE, face‐to‐face group prenatal education; OPE, online asynchronous prenatal education; OR, odds ratio; PE, prenatal education; T1, 10‐23 weeks’ gestation.

a Adjusted for prenatal general anxiety, age, educational attainment, household gross annual income, marital status, and country of birth.

b Tests of model effects on intention were statistically significant for time (Wald χ^2^, 7.46 (df = 1); *P =  *.006) and for PE services (Wald χ^2^, 21.54 (df = 4); *P* <  .001).

### Initiation of Breastfeeding

Our data revealed that 88% of the participants initiated breastfeeding. The logistic regression tests for model effects on breastfeeding initiation were found to be significant for those who had used the study's prenatal education services (Wald, 12.787; *P =  *.01). The indices for initiation of breastfeeding (Table 5) increased significantly as follows: OPE (odds ratio [OR], 6.495; 95% CI, 2.042‐20.662; *P =  *.002), OPE and GPE (OR, 4.913; 95% CI, 1.121‐21.525; *P =  *.04), university education (OR, 3.659; 95% CI, 1.214‐11.025; *P =  *.02); and BSE at T2 (OR, 1.107; 95% CI, 1.063‐1.154; *P* <  .001).

**Table 5:** Determinants of BF Initiation and Exclusivity (Logistic Hierarchical Regression)

	BF Initiation (n = 358)		BF Exclusivity at 4 wk Postpartum (n = 356)	
Determinant	OR (95% CI)	*P* Value	OR (95% CI)	*P* Value
**PE services** ^a^				
No PE (standard prenatal care only)	1 (reference)		1 (reference)	
GPE and OPE (with breastfeeding information)	4.91 (1.12‐21.53)	.035	1.09 (0.48‐2.51)	.675
GPE only (with breastfeeding information)	2.59 (0.25‐26.44)	.254	1.33 (0.29‐6.02)	.713
OPE only (with breastfeeding information)	6.5 (2.04‐20.66)	.002	1.79 (0.86‐3.73)	.118
GPE and OPE (opted out of breastfeeding content)	1.46 (0.57‐3.72)	.43	1.17 (0.55‐2.51)	.675
**General anxiety in late pregnancy (T2)** ^b^	1.11 (0.97‐1.27)	.138	0.95 (0.88 – 1.02)	.171
**Breastfeeding self‐efficacy (T2)** ^c^	1.11 (1.06‐1.15)	<.001	1.04 (1.01‐1.06)	.013
**Parental age, y**				
18‐24	1 (reference)		1 (reference)	
25‐29	1.64 (0.50‐5.39)	.414	0.74 (0.31‐1.77)	.497
30‐34	3.21 (0.77‐13.40)	.111	0.57 (0.22‐1.49)	.253
>35	9.68 (0.83‐113.12)	.07	0.39 (0.13‐1.24)	.11
**Level of education**				
High school or less	1 (reference)		1 (reference)	
College	1.1 (0.38‐3.16)	.864	0.73 (0.3‐1.8)	.497
University	3.66 (1.21‐11.03)	.021	2.28 (1.02‐5.06)	.044
**Gross annual household income**				
<$40,000	1 (reference)		1 (reference)	
$40,000‐$59,000	0.34 (0.06‐1.96)	.229	1.62 (0.54‐4.89)	.393
$60,000‐$79,000	0.63 (0.13‐3.15)	.574	1.49 (0.54‐4.15)	.442
$80,000‐$99,000	0.29 (0.06‐1.44)	.13	1.39 (0.48‐4.01)	.543
≥$100,000	0.66 (0.13‐3.43)	.619	1.49 (0.56‐3.95)	.424
**Marital status**				
Single/separated/divorced	1 (reference)		1 (reference)	
Common law/married	0.83 (0.19‐3.66)	.829	0.95 (0.37‐2.42)	.914
**Country of birth**				
Other than Canada	1 (reference)		1 (reference)	
Canada	2.03 (0.4‐10.24)	.392	0.80 (0.30‐2.13)	.656
**Region of residence**				
A	1 (reference)		1 (reference)	
B	1.54 (0.61‐3.92)	.362	1.39 (0.71‐2.72)	.338

Abbreviations: BF, breastfeeding; df, degree of freedom; GPE, face‐to‐face group prenatal education; OPE, asynchronous online prenatal education; OR, odds ratio; PE, prenatal education; STAI‐6, State‐Trait Anxiety Inventory; T2, 34 weeks’ gestation (date of birth).

a Wald, 12.787; df = 4; and *P =  *.012 for BF initiation; nonsignificant for exclusivity at 4 wk.

b Breastfeeding Self‐Efficacy Score—Short Form, score out of 70.

c STAI‐6, score out of 24.

For nominal sociodemographic variables (region of residence, marital status, and country of birth), the reference group is the one with the lowest number of participants in it. For ordinal sociodemographic variables (parental age, educational level, and household gross annual income), the reference group is the one with the minimal value.

### Exclusive Breastfeeding at 4 Weeks Postpartum

Four weeks after childbirth, 30.9% of participants were exclusively breastfeeding. None of the prenatal education services appeared to be significantly associated with exclusive breastfeeding at 4 weeks postpartum, but the adjusted‐for‐confounding‐variables binomial logistic regression suggests that exclusive breastfeeding was predicted by BSE at T2 (OR, 1.035; 95% CI, 1.007‐1.063; *P =  *.013), and university‐level education (OR, 2.275; 95% CI, 1.024‐5.057; *P =  *.044) (Table 5).

## DISCUSSION

Our findings partially confirm our hypotheses that OPE and GPE with breastfeeding content would be positively associated with breastfeeding‐related outcomes. Participants were more likely to intend to breastfeed and to initiate breastfeeding when exposed to breastfeeding information through both OPE and GPE, compared with standard prenatal care only. Participants who used OPE were more likely to have breastfeeding intentions and initiation. Although prenatal BSE was positively associated with breastfeeding initiation and exclusivity, neither GPE nor OPE, alone or in combination, affected BSE in late pregnancy.

It is important to point out that in both OPE and GPE services, breastfeeding was presented as a matter of personal choice. The advantages and disadvantages of various infant feeding methods were described in OPE and GPE to enable parents to make informed decisions. A substantial proportion of our sample (29%) opted to use OPE or GPE without accessing the breastfeeding content, which exceeds that of those who did not intend to breastfeed at T1 (21.5%) or T2 (15.5%). This suggests that some participants with breastfeeding intentions may have sought alternative information or felt no need for breastfeeding guidance.

Our findings reveal a positive association between engagement with OPE breastfeeding information and reported breastfeeding intentions. This is in line with a study that evaluated the influence of OPE on breastfeeding^44^ in which 14 of the 21 participants who were pregnant reported that OPE had a positive impact on their breastfeeding intentions. OPE, although in a virtual live format, also increased breastfeeding intentions of a sample of 156 pregnant persons during the COVID‐19 pandemic.^44^ In both studies, some participants reported prior breastfeeding experience, whereas our sample consisted of women who were nulliparous. Beyond differences in our respective interventions and populations, the studies’ designs do not allow us to conclude that OPE is an effective strategy for consolidating breastfeeding intentions in either nulliparous or primiparous pregnant persons.

Studying a real‐world intervention in which the targeted population is not entirely inclined toward breastfeeding provides valuable insight into the current literature. Previous intervention studies displaying initiation rates of 100% included only participants who intended to breastfeed,^45^, ^46^ which have been positively associated with breastfeeding‐related decisions.^7^, ^38^ OPE was not exclusively targeting breastfeeding outcomes, yet it was nevertheless associated with an increased likelihood of initiating breastfeeding. However, our analyses show that using GPE services alone, in addition to standard prenatal care, was not associated with increased breastfeeding intention, initiation, or exclusivity. This differs from a previous cohort study, in which those enrolled in a GPE course with 2.5 hours of breastfeeding education were significantly more likely to initiate breastfeeding than matched nonparticipants (93.65% vs 87.48%; χ^2^ (1), 9.077; *P =  *.003).^20^ The lack of studies evaluating how GPE (alone or with OPE) influences breastfeeding intentions in real‐life settings hampers comparison with our results.

Our results do not support our hypothesis that knowledge and skills developed in prenatal education classes would improve BSE precursors. This might be linked to the fact that interventions did not provide model/theory‐based breastfeeding education, for which evidence suggests increased BSE.^12^ Neither GPE nor OPE, alone or in combination, affected BSE in late pregnancy; however, prenatal BSE was positively associated with breastfeeding initiation and exclusivity. Again, the GPE and OPE curriculum design, intended to provide the requisite information (eg, the OPE scientifically validated content) to make an informed choice on various perinatal topics must be considered when comparing our results with the prenatal BSE measured in other studies. Moreover, the number and duration of GPE sessions as well as the educators themselves varied across sites. In contrast, OPE was standardized, although participants were free to focus on topics of their choice. In an experimental study of the effects of adding a breastfeeding booklet and 2 breastfeeding GPE sessions of 60 to 90 minutes to routine care in a sample of 130 primiparous and multiparous pregnant persons, researchers observed a higher prenatal BSE in pregnant persons exposed to the intervention compared with those who received only the routine prenatal care (mean 70.84 [SD, 8.68] vs 55.02 [SD, 9.49], respectively; *P* <  .001).^47^ Given that their groups were controlled for previous pregnancy and breastfeeding experience, it is reasonable to conclude that the sessions' emphasis on breastfeeding contributed to this result.

Although our study's breastfeeding initiation rate (88%) was similar to reported data for Québec before the COVID‐19 pandemic (88.9%),^48^ 30.9% of participants exclusively breastfed their newborn at 4 weeks postpartum. We are aware that our findings correspond to regional data; these rates are nonetheless considerably lower than those reported prior to the pandemic for exclusive breastfeeding at 4 weeks in Canada (72.8%).^48^ This is surprising and requires further investigation because our sample's sociodemographic factors, such as higher educational level and household income, are usually associated with better breastfeeding outcomes.^14^ Considering these suboptimal rates, as well as current breastfeeding rates in North America,^5^, ^6^ it is important to identify other factors that may help predict longer exclusive breastfeeding.

Our findings, in line with Dennis’ theoretical assumptions,^16^ suggest that prenatal BSE can be used as a predictor of exclusive breastfeeding among women who are nulliparous. Accordingly, it is likely that BSE theory^16^ and measures^28^, ^49^ may be useful for informing supportive interventions, especially when nurses or midwives identify a low level of breastfeeding intentions during pregnancy. More attention could be paid to prenatal knowledge and attitudes regarding breastfeeding because these are other modifiable psychosocial factors reported to influence breastfeeding intentions.^8^, ^13^ Incorporating activities to enhance expectant and new parents’ BSE would benefit the development of prenatal education curricula because theory‐based prenatal interventions are more likely to improve breastfeeding outcomes.^12^, ^17^, ^50^ In addition, professional support for breastfeeding after childbirth,^35^ as well as endeavors to ensure that breastfeeding‐related sections of the OPE website are appealing and that community‐based organizations are recognized and used by new parents, could have a positive influence on breastfeeding outcomes.^19^, ^51^

This study has several limitations. Our findings should be interpreted with caution because the study design does not imply cause‐and‐effect relations between the prenatal education services, BSE, and underlying breastfeeding behaviors. All participants may have had access to other sources of breastfeeding information, the use of which could be closely monitored in further research. Participants were recruited by convenience, potentially introducing selection bias. Because our study was not experimental, no participant was assigned to a specific prenatal education service and remained free to use or decline the services offered. Additionally, they could choose whether to receive information about breastfeeding. This led to an uneven distribution of participants that might affect the power/stability of the analysis. Only 23% of participants used the GPE services (either alone or with OPE). Although this may suggest that in‐person group services are not as essential as previously assumed, it raises the hypothesis that using both OPE and GPE is associated with greater motivation to breastfeed and to seek as much prenatal information as possible. With the emergence of live online prenatal education formats prompted by the COVID‐19 pandemic, more research is needed to assess their sustainability and effectiveness in improving prenatal education services utilization rates among nulliparous. Finally, the study sample is drawn from 2 specific regions, which may limit generalizability to other areas or countries.

Notwithstanding these limitations, our study has strengths. It contributes to the scant literature comparing OPE and traditional in‐person GPE services.^52^ The prospective cohort study design enabled longitudinal data collection in a pre–COVID‐19 context, thereby limiting potential biases related to the public health crisis itself. Our findings can inform perinatal nurses, midwives, community organizations, and other health care professionals of the potential advantages of different prenatal educational modalities on the breastfeeding intentions and choices of nulliparous women. It can also be useful for government organizations that have decision‐making power over the content and format of prenatal education offerings to make choices that help achieve their breastfeeding targets.

## CONCLUSION

Our study highlights the complementary contributions of OPE and traditional face‐to‐face GPE services to breastfeeding. Although exposure to the breastfeeding content of either OPE alone or OPE combined with GPE was associated with first‐time parents’ breastfeeding intentions and initiation, none of these interventions appeared to influence their prenatal BSE or breastfeeding exclusivity at 4 weeks postpartum. Knowing that women who are nulliparous chose OPE over GPE can help policy makers identify potential avenues to strengthen equity in the integration of prenatal breastfeeding education programs into the perinatal care continuum. This, along with interventions targeting BSE, could provide expectant parents with better strategies for choosing and maintaining breastfeeding.

## CONFLICT OF INTEREST

No conflict of interest to declare.

## Supporting information




**Appendix S1**. Strengthening the Reporting of Observational Studies in Epidemiology (STROBE) checklist.

**Appendix S2**. Prenatal education services
