# Supplementary material for: The Effectiveness of Online Versus Group Prenatal Education on Breastfeeding Determinants and Outcomes in Nulliparous Women: A Prospective Cohort Study
Source: J Midwifery Womens Health. 2026 Jul 7;71(5):771–81. doi: 10.1111/jmwh.70156 (PMC13655187; doi:10.1111/jmwh.70156)
Supplement: Supplementary file 1 [file JMWH-71-771-s001.docx]

**Supplementary Information : Appendix S2. Description of Interventions According to the Template for Intervention Description and Replication (Hoffmann et al., 2014).**

| **TIDieR Items** | **Group Prenatal Education Services (GPE)** | | **Online Prenatal Education Services (OPE)** |
| --- | --- | --- | --- |
|  | **Health and Social Services Institution A** | **Health and Social Services Institution B** | **OPE** |
| **Item 1: *Brief name*** | In-person group prenatal sessions/classes | In-person group prenatal sessions/classes | Presented as a reliable and scientifically validated source of information, offered by institutions A and B as of April 2018 |
| **Item 2: *Why*** | Goal**:** To help families expecting a child stay healthy—physically, mentally, and emotionally and to give the information and support needed to make this a happy, harmonious time.  Objectives: To foster the acquisition of knowledge and the development of skills, to prevent the emergence or deterioration of problematic situations and to reduce anxiety regarding pregnancy, labor, birth and newborn care. To harmonize practices surrounding prenatal sessions, both in terms of content and modalities of use. To prepare parents for pregnancy, birth and the first days of their child's life. To answer parents’ main questions during pregnancy.  To offer an opportunity to meet other couples and share experiences. | Goal/objectives: To assist expectant parents in optimizing their pregnancy experience, preparing for the arrival of their child and navigating the first days as new parents. To provide comprehensive guidance addressing the primary queries of prospective parents during pregnancy and facilitate networking opportunities for attendees. | The OPE program began in January 2009, 19 months after it was created. It was added to the A and B institutions service offer in April 2018.  It is a web-based resource that focuses on child development. Information is available on a timeline that tracks the child's development (i.e., pregnancy, 0 to 12 months, etc.).  Mission: Support parents in Quebec in fostering their child's development from conception to age 8.  Funding: Funded by a philanthropic company.  No profit-making, commercial attachments, financial conflicts of interest, or advertising. |
| **Item 3: *What (materials)*** | List of equipment used during in-person prenatal sessions:  o Computer  o Screen  o Projector  o USB key  o Pencils  o Practical guide with scientifically supported information  o Attendance sheet  o Removable pelvis  o Fabric newborn and placenta  o Fabric breast  o Ice-breaker activity supplies | No standardized material (e.g., PPT, guide) | Web pages  o User information sheets (with the option of reading aloud)  o Blogs  o Videos  o Infographics  o Photos  o Podcasts  Newsletter (via email) |
| **Item 4: *What (procedures)*** | The GPE offer was presented at the routine pregnancy follow-up meeting with the nurse. Parents may also access the same information on the institution’s website. | The GPE offer was presented at the CLSC regular pregnancy follow-up for all pregnant persons in the region. | A bookmark about the OPE was included in the free pregnancy/early childhood practical guide distributed to parents-to-be during the pregnancy follow-up. The website is organized by age group. The suggested user information sheets are based on keywords. Each card has metadata and matches keywords to display relevant content. The content is varied and changes according to the person. The interface was designed to make content accessible to people with an 11th-grade literacy level. This resulted in uncluttered pages, highlighted headings, a special font size, an A-to-Z index, a search engine, an instant glossary, and simplified navigation. Three rounds of usability testing were done with different groups to improve navigation and make sure the information is relevant to parents. The team can be contacted through forms that are available on the website. |
| **Item 5: *Who provided*** | The CLSC (local community service centers) perinatal registered nurses.  Session #4 offered in collaboration with a breastfeeding support group counselor. | The CLSC perinatal registered nurses.  10/16 of the service points involve non-profit organizations to cover various topics. | All published articles are subject to scientific review. The name of the person who reviewed the page and their profession is shown at the bottom of each page, along with the month and year it was last updated.  The team includes a director, a web and marketing manager, an editor-in-chief, 2 desk officers, 2 web developers, one content and social media coordinator, and a customer service agent. In addition, the team employs approximately 20 editors. |
| **Item 6: *How*** | The in-person intervention is designed for groups of up to 20 to 24 participants. The number of participating couples is typically between 12 and 15 in metropolitan areas and between 5 and 6 in rural areas. | In-person group sessions | Web pages: The website can be accessed directly without human interaction, allowing users to navigate it at their own pace, independently or with others (i.e., partner).  The newsletter is distributed via email on a weekly basis (registration required). |
| **Item 7: *Where*** | 9 service points (8 in CLSCs and 1 in a birth center) in 3 areas | 15 service points (all in CLSCs, except for one birth center) in 4 areas | Anyone can access the website via the URL or an online search. No account is required. |
| **Item 8:**  ***When and how much*** | One session per week for 4 consecutive weeks. Classes are held in the evening (6:30 to 8:30 p.m.), or on Saturday mornings.  Maximum cost $50 (depending on the needs of participants) | The number of sessions and duration may vary from 2 to 6 sessions and two and a half hours, respectively.  The time allocated to each theme may vary.  Free of charge | This information resource provides asynchronous distribution, ensuring that information is always available. A personalized newsletter service is available to users who subscribe via email.  Free of charge |
| **Item 9:**  ***Tailoring*** | N/A | N/A | Upon subscribing to the newsletter, users may indicate the number of children for whom they wish to receive communications. One newsletter is sent per child, personalized with the child's birthday, on a weekly basis. The website's home page features a random dynamic section, and a section managed by site administrators, including contests, news, and other updates. The website does not utilize personalized connections or cookies, so each visit is treated as a new session. |
| **Item 10:**  ***Modifications*** | * Same PowerPoint presentation used at all service points  ** Breastfeeding session now available at all service points in collaboration with local breastfeeding support group  *** Number of service points reduced from 9 to 8. | *In some CLSCs, the number of sessions was changed from 2 to 4.  **The session content was adjusted according to the knowledge questionnaire of the cohort study (anonymized for submission). | The website is regularly updated to reflect developments in the scientific literature and current events. The site's home page features news headlines, podcasts, recently added articles, and more. The website is therefore constantly evolving.  Two people are responsible for updating the content and will go back through an entire topic to update the references. They do this on an ongoing basis. Occasionally, they may focus on a particular topic if it has recently received particular attention in the media or in research. |
| **Item 11:**  ***How well (planned)*** | The intervention was already implemented when the cohort study began. A report on health workers' practices and mothers' and fathers' needs regarding prenatal information was issued. However, there was no evaluation of compliance with the planned curriculum. | The intervention was already implemented when the cohort study began. There is a questionnaire to assess participants' satisfaction with the classes, but no evaluation of compliance with the planned curriculum. | N/A |
| **Item 12:**  ***How well (actual)*** | GPE services were interrupted due to the emergency state resulting from the COVID-19 pandemic in March 2020. This interruption remained in place at the conclusion of the data collection period in September 2020. | | N/A |

**References (Appendix S2)**

Hoffmann TC, Glasziou PP, Boutron I, et al. Better reporting of interventions: template for intervention description and replication (TIDieR) checklist and guide. *BMJ*. 2014;348:g1687. doi:10.1136/bmj.g1687
